# Bilateral hearing loss as an initial presentation of Creutzfeldt-Jakob disease

**DOI:** 10.1590/1980-57642021dn15-040016

**Published:** 2021

**Authors:** Janaina Mariana de Araujo Miranda Brito-Marques, Eduardo Sousa de Melo, Fabíola Lys de Medeiros, Cristiano Sobral de Carvalho, Paulo Roberto de Brito-Marques

**Affiliations:** 1 Department of Neurology, Hospital Universitário Oswaldo Cruz, Universidade de Pernambuco – Recife, PE, Brazil.; 2 Department of Neuropsychiatry, Unit of Neurology and Neurosurgery, Universidade Federal de Pernambuco – Recife, PE, Brazil.; 3 Medical Sciences College, University of Pernambuco, Recife, PE, Brazil.

**Keywords:** dementia, prion disease, hypoacusis, demência, doença de príon, hypoacusis

## Abstract

We reported a case of a 61-year-old male patient with anacusis, cerebellar syndrome, myoclonus, and frontal signs. The brain magnetic resonance imaging showed bilateral striated hyperintensity of the fluid-attenuated inversion recovery and restricted diffusion in the diffusion-weighted imaging and hypointense areas corresponding to the apparent diffusion coefficient in the cerebral cortex. The autopsy revealed positive immunohistochemistry for the PrPSc protein. Creutzfeldt–Jakob disease presenting with hearing loss is unusual.

## RESUMO.

Relatamos o caso de um paciente do sexo masculino, 61 anos, com anacusia, síndrome cerebelar, mioclonia e sinais frontais. A ressonância magnética cerebral mostrou hiperintensidade estriada bilateral do *fluid-attenuated inversion recovery* (FLAIR) e difusão restrita no *diffusion-weighted imaging* (DWI) e áreas hipointensas correspondendo ao coeficiente de difusão aparente no córtex cerebral. A autópsia revelou imuno-histoquímica positiva para a proteína PrPSc. A doença de Creutzfeldt-Jakob que se apresenta com perda auditiva é incomum.

**Figure 1.**
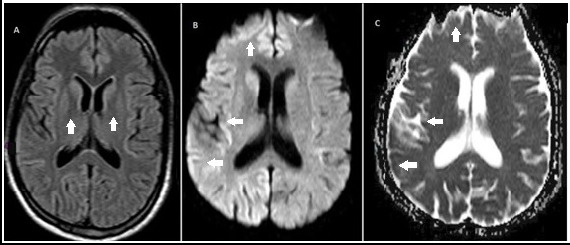
Brain MRI. Panel A show hyperintensity in bilateral striatum in the FLAIR sequence. Panels B and C shows water restriction in cortex with a predominance in the right hemisphere (DWi/ADC map).

**Figure 2.**
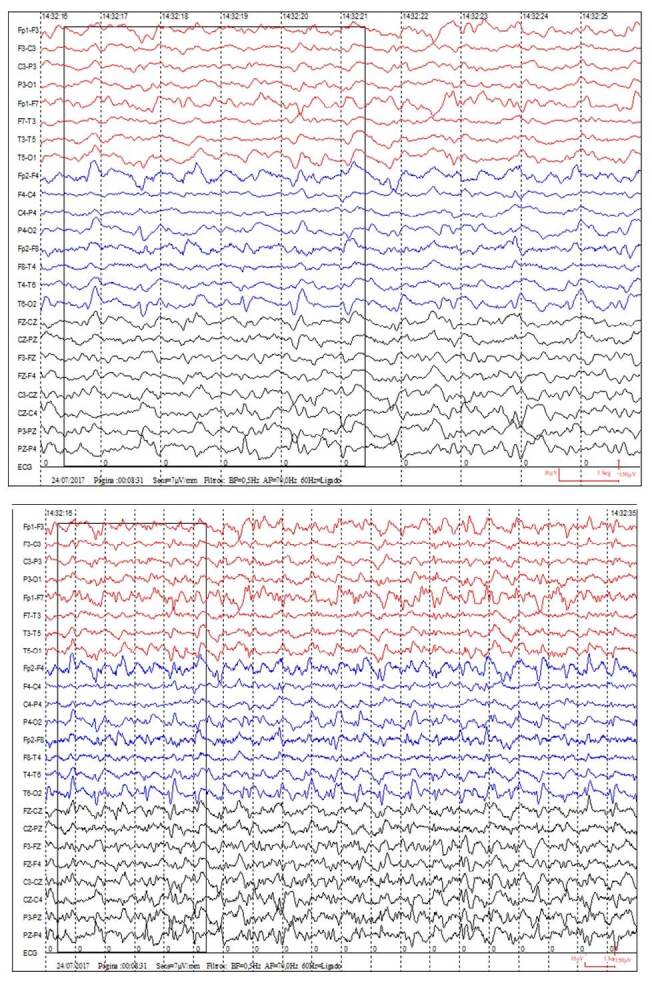
Electroencephalogram with periodic activity, biphasic or triphasic morphology, of 0.8–1.3 seconds, with diffuse distribution, lateralized to the right cerebral hemisphere, and predominantly posterior.

A 61-year-old man presented with progressive bilateral hearing loss, and gait disturbance for 2 months. On examination, he presented anacusis, cerebellar syndrome, myoclonus, and frontal signs. Brain magnetic resonance imaging (MRI) showed bilateral striatum fluid-attenuated inversion recovery hyperintensity and restricted diffusion on diffusion-weighted imaging and corresponding apparent diffusion coefficient hypointense areas in the cerebral cortex (Figure 1). The electroencephalogram (Figure 2) showed three-phase periodic activity (0.8–1.3 Hz). Cerebrospinal fluid was in range, tau protein was 440 ng/L (slightly elevated), and 14-3-3 protein was negative. Auditory evoked potential revealed severe bilateral dysfunction of the vestibulocochlear nerves. In addition, laboratory tests, anti-thyroid peroxidase (TPO) test, autoimmune tests, and paraneoplastic screening were performed with negative results. The patient developed progressive cognitive worsening, akinetic mutism, and cortical blindness. After 5 weeks in hospital, the patient developed aspiration pneumonia progressing to sepsis and death. The pathology with immunohistochemistry was positive for PrPSc protein.

In the literature, few cases of bilateral hearing loss have been reported as an initial symptom of Creutzfeldt-Jakob disease (CJD). In our review, these cases were evaluated with audiometry and/or auditory evoked potentials. In only one of the cases, the auditory evoked potentials showed a central pattern of hearing loss. Two cases, including ours, had vestibulocochlear pattern involvement, another case was normal, and in the remaining cases, it was not possible to carry out the test or if the result was inconclusive.^1,2,3,4,5,6^

Other causes of hearing loss associated with progressive encephalopathy should be taken under consideration, such as Whipple’s disease, Lyme disease, human immunodeficiency virus, Susac disease, sarcoidosis, central nervous system vasculitis, lymphoma, and paraneoplastic disease.^4^

Prion disease should be considered in cases of rapidly progressive dementia with myoclonus; however, in one-third of the cases, it may initially manifest with atypical symptoms such as aphasia, pure ataxia, and visual or hearing loss. In atypical cases, the recognition of typical patterns for CJD on the brain is MRI and is of great importance to guide for further investigation. [5,6] Despite of bilateral hearing loss being a rare form of initial presentation of CJD, it should be part of the differential diagnosis of progressive encephalopathies with auditory symptoms.
